# Development of a UK Online 24-h Dietary Assessment Tool: myfood24

**DOI:** 10.3390/nu7064016

**Published:** 2015-05-27

**Authors:** Michelle C. Carter, Salwa A. Albar, Michelle A. Morris, Umme Z. Mulla, Neil Hancock, Charlotte E. Evans, Nisreen A. Alwan, Darren C. Greenwood, Laura J. Hardie, Gary S. Frost, Petra A. Wark, Janet E. Cade

**Affiliations:** 1 Nutritional Epidemiology Group, School of Food Science and Nutrition, University of Leeds, Leeds LS2 9JT, UK; E-Mails: ml09saa@leeds.ac.uk (S.A.A.); m.morris@leeds.ac.uk (M.A.M.); n.hancock@leeds.ac.uk (N.H.); c.e.l.evans@leeds.ac.uk (C.E.E.); n.a.alwan@soton.ac.uk (N.A.A.); j.e.cade@leeds.ac.uk (J.E.C.); 2 School of Food Science and Nutrition, King Abdul-Aziz University, PO Box 42807, 21551 Jeddah, Saudi Arabia; 3 Centre for Spatial Analysis and Policy, School of Geography, University of Leeds, Leeds LS2 9JT, UK; 4 Global eHealth Unit, Department of Primary Care and Public Health, London School of Public Health, Imperial College London, London W6 8RP, UK; E-Mails: zeinab.mulla@imperial.ac.uk (U.Z.M.); p.wark@imperial.ac.uk (P.A.W.); 5 Academic Unit of Primary Care and Population Sciences, Faculty of Medicine, University of Southampton, Southampton General Hospital, Southampton SO16 6YD, UK; 6 Division of Biostatistics, Leeds Institute of Genetics, Health and Therapeutics, University of Leeds, Leeds LS2 9JT, UK; E-Mail: d.c.greenwood@leeds.ac.uk; 7 Molecular Epidemiology Unit, Leeds Institute of Genetics, Health and Therapeutics, University of Leeds, Leeds LS2 9JT, UK; E-Mail: l.j.hardie@leeds.ac.uk; 8 Nutrition and Dietetic Research Group, Department of Investigative Medicine, Hammersmith Hospital, Imperial College London, London W12 0NN, UK; E-Mail: g.frost@imperial.ac.uk

**Keywords:** dietary assessment, 24-h dietary recall, nutrition assessment

## Abstract

Assessment of diet in large epidemiological studies can be costly and time consuming. An automated dietary assessment system could potentially reduce researcher burden by automatically coding food records. myfood24 (Measure Your Food on One Day) an online 24-h dietary assessment tool (with the flexibility to be used for multiple 24 h-dietary recalls or as a food diary), has been developed for use in the UK population. Development of myfood24 was a multi-stage process. Focus groups conducted with three age groups, adolescents (11–18 years) (*n =* 28), adults (19–64 years) (*n =* 24) and older adults (≥65 years) (*n =* 5) informed the development of the tool, and usability testing was conducted with beta (adolescents *n =* 14, adults *n =* 8, older adults *n =* 1) and live (adolescents *n =* 70, adults *n =* 20, older adults *n =* 4) versions. Median system usability scale (SUS) scores (measured on a scale of 0–100) in adolescents and adults were marginal for the beta version (adolescents median SUS = 66, interquartile range (IQR) = 20; adults median SUS = 68, IQR = 40) and good for the live version (adolescents median SUS = 73, IQR = 22; adults median SUS = 80, IQR = 25). Myfood24 is the first online 24-h dietary recall tool for use with different age groups in the UK. Usability testing indicates that myfood24 is suitable for use in UK adolescents and adults.

## 1. Introduction

Reliable assessments of the associations between diet and health require estimation of usual diet. Traditional methods of dietary assessment such as multiple 24-h dietary recall interviews and food diaries can be impractical for large cohort studies often requiring costly and time-consuming manual nutrition coding. Food frequency questionnaires (FFQs) have been used in epidemiological studies due to their relative ease of administration and low participant burden. However, FFQs are subject to measurement error due to imprecision with respect to portion sizes, limited food lists, lack of detail regarding food preparation and the potential for misclassification of participants according to intake [1,2]. Multiple 24-h dietary recalls effectively represent habitual dietary intake and have shown less bias in reporting of energy and protein intakes when compared with FFQs using biomarker measures [3]. Along with convenience and scalability, incorporation of an online 24h dietary recall into large prospective cohort studies may advance our understanding of the nutritional determinants of disease [1] through possibly improved assessment of diet. Ultimately, this would allow for more reliable evidence-based formulations of health policies.

A number of online dietary assessment systems have already been developed [4,5,6,7,8,9]. In the United States, Subar *et al.* (2010) [10] have developed ASA24 (Automated Self-Administered 24 h Recall), which is currently being used in many studies. ASA24 is based on the USDA’s “Automated Multiple Pass Method” (AMPM) [11], which involves recording intake in a series of defined “passes” to elicit a detailed recall. The AMPM has been validated against doubly-labeled water and shown to accurately estimate mean total energy intake in “normal”-weight individuals [12].

While an online 24-h dietary checklist for the UK exists (the Oxford WebQ [9]), there is currently no automated 24-h recall dietary assessment tool appropriate for the UK population. To address this gap, a fully automated online 24-h dietary assessment system, myfood24 (Measure Your Food on One Day) was developed with the flexibility to be self- or interviewer-administered as required and to be used as either a 24-h dietary recall or a food diary. This paper aims to describe the myfood24 development process and provide an overview of its features and functionality relating to self-administered use.

## 2. Experimental Section

**Figure 1:**
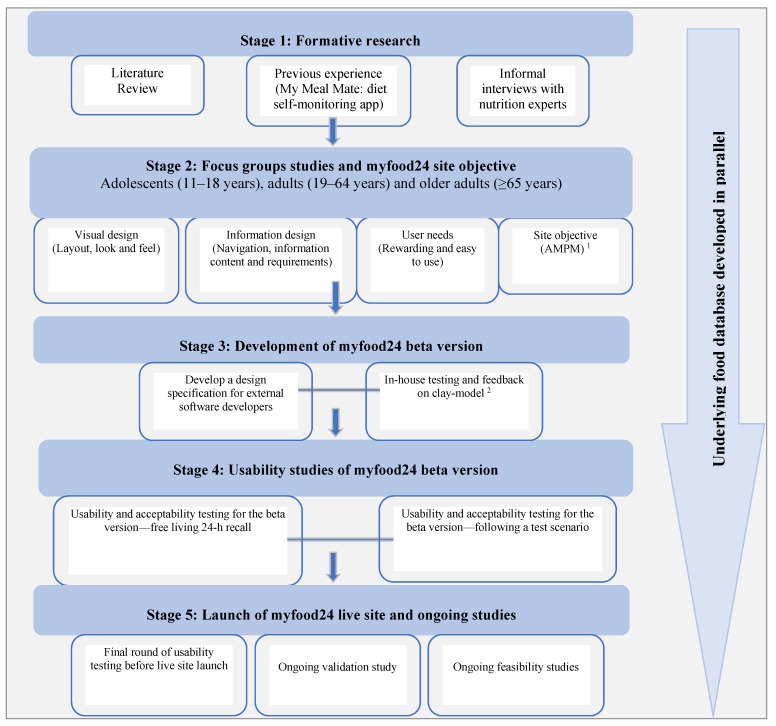
Flow chart illustrating the development process of myfood24. ^1^ AMPM = Automated Multiple-Pass Method; ^2^ “Clay model” = static clickable wire-frame without database functionality.

Development of myfood24 was a multi-stage process, as summarized in Figure 1. Features of myfood24 are described in Table 1. Methods and results are presented together by stage of the project. Results comprise both qualitative and quantitative data. Ethical approval for this work was provided by the University of Leeds Research Ethics Committee (reference number MEEC 11-146).

**Table 1:** Features of myfood24.

Participant Area	Researcher Area
“Getting started” instructions displayed on first screen Search function (with options to filter by category or brand) “Make a List” searching (an optional small area to list everything consumed with free text. Once completed, the search function works its way through this list. The participant is then able to add individual items remembered afterwards) Portion size selection area (including photos; standard pack sizes; enter own amount) Recipe Builder (user is able to search and log ingredient combinations as individual recipes in a separate section) Recently used items to allow quick entry of repeated foods/drinks Food lists by meal (with time of meal optional for researcher) Drag-and-drop between meals Prompt for commonly missed accompaniments for a number of items (e.g., milk with cereal, spread on bread) Final review screen encouraging user to check before submission. Includes list of commonly forgotten food items. Supplementary questions (e.g., did you take any vitamins, minerals or other supplements during your day? Was the food consumed representative of a typical day?—Optional to researcher) Login area where the participant can select which recall day to complete Help—(including a specific area of the site with detailed help text, mouseovers/hover text over specific parts of the website and help videos) Nutrient feedback (energy, protein, fat, carbohydrate, fiber, and salt—Optional to researcher).	Customization Add project specific text and logo Tailored additional help text (in addition to default help text) Tailored invitation and reminder emails to participants (how many and how frequently) Tailored optional “thank you” email to go out at the end of the study Select recall or diary option Select whether to record time of meals or not Option to display nutrient summary details to participant or not Supplementary questions (optional). Study participant management—upload email addresses; send automatic reminders at specified dates “Take control” function for interviewer mode (The researcher is able to use this button to access the tool and complete and submit the recall/diary on the participants behalf) Export of summary macronutrient and micronutrient analysis output (from potential 120 nutrients) to a CSV file Export of detailed food and nutrient analysis (120 nutrients) output to a CSV file

## 3. Results

### 3.1. Stage 1: Formative Research

**Figure 2:**
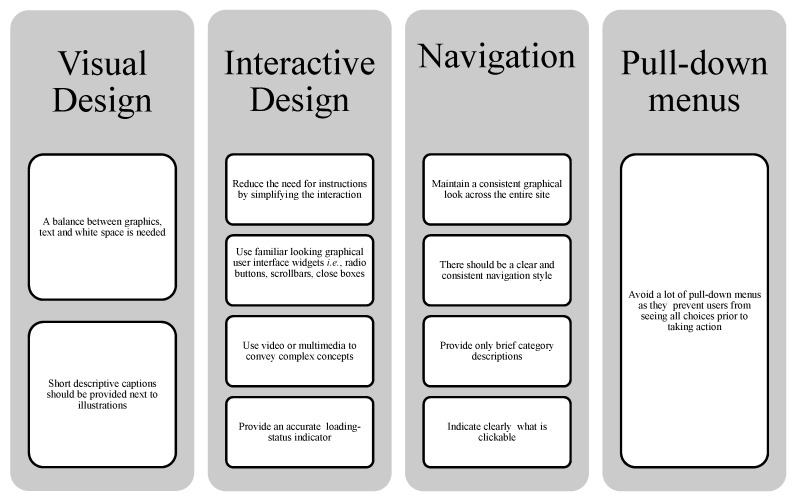
Key requirements to be considered in website design identified from the literature [21,22,23,24,25,26].

In preparation for the development of myfood24, substantial formative research was conducted by reviewing the literature on existing computerized dietary assessment tools and discussion with experts in the field of dietary assessment. Consideration was also given to factors which enhance usability and engagement with websites in general (Figure 2). The findings were used to inform the focus groups among all ages in Stage 2 and in particular to focus on the design of the “food search” and “portion size estimation” components of the system. Several existing dietary assessment tools from other countries were inspected and presented to focus group participants in order to aid discussion; these included: ASA24 (Automated Self-Administered 24-hour Recall) [5], DietDay [4] and NutriNet-Santé [6].

Several methods for finding foods have been employed in existing online dietary assessment tools. The food list in ASA24 [5] is hierarchically organized into food categories, with a free-search option. Young Adolescents’ Nutrition Assessment on Computer (YANA-C) [13] uses a “tree” structure (if there are no matches, the closest food must be selected), and others like DietDay [4] have a “fast track” option, which allows the respondent to save commonly-consumed foods. Standard food portion sizes have been used in several tools, such as Synchronised Nutrition and Activity Program™ (SNAP™) [14], Oxford WebQ [9], and Reality [15], while others like ASA24 [5] and NutriNet-Santé [6] have used portion size images to guide selection of appropriate portion sizes.

Experience was drawn upon from the previous development of a smartphone app supporting self-monitoring of diet for weight loss, “My Meal Mate” (MMM) [16]. In the pilot trial of MMM, the food composition database was found to be a limiting factor in engaging with the app, and participants struggled to find the correct food and drink items. With this in mind, we created an extensive new UK food composition database for incorporation into the tool. The database currently includes ~45,000 UK branded and generic foods with their associated pack and portion sizes (by comparison, the existing British food composition tables contain ~3500 generic food items) [17].

In the new database, 5669 food items contain portion images [18]. Portion images were added for the 100 most commonly consumed food types, and for all other foods for which they were relevant. For example, the image for sliced chicken breast was also applied to other white sliced meats, such as turkey and pork. Each food type with associated portion images has the option for the user to select from seven portion sizes images. The foods with portion size images are the top 100 foods in terms of frequency of consumption, weight of consumption and contribution to energy intake, identified from data collected during the National Diet and Nutrition Survey conducted with young people (4–18 years) [19]. All the food items in the database have been mapped via their back-of-pack nutrient information to the McCance and Widdowson composition of food codes [17]. Details of the development of the food database are reported elsewhere [20].

### 3.2. Stage 2: Focus Groups

The findings of Stage 1 were used to inform the focus group questions among all ages in Stage 2, in particular, to focus on the design of the “food search” and “portion size estimation” components of the system. Focus groups were conducted with adolescents, adults and older adults to understand what features people might prefer in an online dietary assessment tool and whether or not they might use such a tool. These discussions were <60 min in duration, facilitated by a moderator and assistant moderator, audiotaped and transcribed *verbatim* alongside field notes taken by a research assistant. Group discussions were allowed to flow naturally but were divided into semi-structured sections with prompts on the following topics: preference for how to search a food composition database; preferences for estimating portion size; lay-out; usability features; potential incentives for use; and the maximum amount of time that individuals would be willing to spend using the system.

Adolescent participants were recruited by email and posters from two secondary schools in Leeds. Adults were recruited by email and posters advertising the study to staff at the University of Leeds and older people were recruited by contacting the Leeds branch of the University of the Third Age (an international educational organization aimed at retired people).

Data were analyzed using a basic thematic analysis. Findings were organized according to predefined topics (linked to the website design and key functions) and not data-derived themes, as the data were used to inform the website specification. Results are summarized in Table 2. All age groups preferred images to aid portion size estimation and a clean design with no “pop-ups”. Whereas adults and older adults were prepared to spend a bit longer completing the tool, adolescents were unwilling to spend more than 15 min. All expressed a desire for feedback on their nutritional intake.

**Table 2:** Summary of feedback from focus groups on preferred options for an online dietary assessment tool, by age and discussion topic on which views were sought.

	Adolescents (11–18 Years) (*n =* 28)	Adults (19–64 Years) (*n =* 24)	Older Adults (≥65 Years) (*n =* 5)
**Summary characteristics**			
**Mean age years (SD)**	14 (2)	36 (13)	67 (3)
**Male, %**	61	37	40
**Topics on which views were sought during focus groups**			
	**Focus group feedback on topics discussed**		
Database searching	Keyword or category preferred Predictive text useful	Keyword preferred Speed important Concerns expressed about long lists	Keyword preferred Speed important Concerns expressed about long lists
Portion size estimation	Images desired Variety of options (e.g., plate, packet) preferred	Images desired Manual input from packaging useful	Images desired Portion size plate image suggested
Layout	Balance between text and images important No pop-ups No “childish” design or colors	Uncluttered look desired Health neutral in terms of color and design No pop-ups	Uncluttered look desired No pop-ups
Usability features/support	10–15 min for completion, maximum 15 min	10–20 min acceptable for completion but depends on required frequency of use Recipe function “Frequently used” function Typical day indicator	20–30 min acceptable completion time, up to 60 min if infrequent
Help option	Trial and error preferred; short video or avatar helpful (only young adolescents)	Hover features; FAQs; no avatar or video	Paper based written wanted. Help by telephone support desirable
Incentives	Feedback on intake and guidance on improving diet preferred Cash desired	General report on intake desired	General report on intake desired Incentive enough to benefit the overall study

### 3.3. Stage 3: Development of myfood24 Beta-Version

#### 3.3.1. Develop a Design Specification

**Figure 3:**
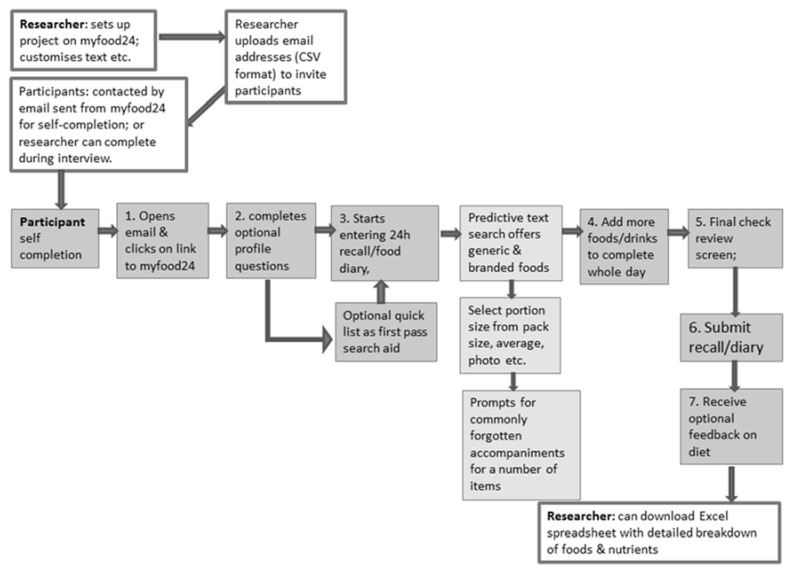
Typical myfood24 workflow for researcher and participant (shaded boxes represent participant actions; unshaded boxes represent researcher actions).

Using results of the initial focus groups, the design specification was developed in collaboration with software developers and the myfood24 project consortium. The myfood24 consortium combines researchers from the University of Leeds and Imperial College London. Members of the consortium bring a wide range of expertise, including nutritional epidemiology, dietetics, dietary assessment, public health, biostatistics and molecular epidemiology. The two major sections for development were the participant area and the researcher area of the website. The researcher area was required so that projects could be set up and data extracted with ease. A typical example of workflow using myfood24 is displayed in Figure 3.

#### 3.3.2. In House Testing/Feedback on “Clay Model”

The first prototype of myfood24 was a static “clay model” without database searching functionality (individual wireframes with the ability to click-through the screens to understand the navigation) (Figure S1a, b in supplementary materials). It was subjected to in-house testing rather than external user testing, as it was not yet deemed to be at the required standard for external users. The clay model feedback informed the development process of the beta model. An iterative development approach was adopted throughout the project, the project team as well as a small number of employees and students of the University of Leeds were involved in user testing and feedback to the software developers at regular intervals.

### 3.4. Stage 4: Usability Testing of the Beta Version

User testing took place using the beta version of the software. This involved using myfood24 to self-complete a 24-h recall. The beta version of myfood24 only contained a sample of items from the food composition database. Fourteen adolescents, eight adults and one older person were involved in testing the beta version of myfood24. There is debate about the sample size needed to identify usability problems, but it has been reported that 80% of usability problems are uncovered with the inclusion of around five participants [27,28]. These studies also show diminishing returns in uncovering problems as sample size increases [27,28]. Questionnaires were administered to gather data on user demographic characteristics, usability (using the Systems Usability Scale (SUS)) and confidence in using technology. The SUS is a 10-item scale with users asked to rate their level of agreement with 10 usability statements (1 = strongly disagree; 5 = strongly agree), which gives an overall usability score from 0 to 100 [29]. In general, a product with an SUS of 70 is considered to be good and products with less than a score of 70 would be judged as marginal. Products with a score of less than 50 are considered to be a cause for concern [30]. Participants were also asked to self-rate their confidence in using technology on a Likert scale of 1 to 10. The key issues identified with the beta version of myfood24 during usability testing are presented in Table 3, and all reported issues were fed back to the software developers to inform the final phase of development. The sample characteristics, SUS score and self-rated technology confidence score of those who conducted usability testing of the beta and live version of myfood24 can be found in Table 4.

**Table 3:** Key issues identified with the beta version of myfood24 during usability testing.

Problem Identified	Improvements Made to myfood24
List of foods presented after database search appeared to confuse users. This was because brand and generic items were mixed	Generic items were displayed first in the search list
Users could not find foods if they misspelt them (e.g., zucchini, avocado and baguette)	A large database of misspellings and synonyms was created to improve searching the underlying food composition database
Problems in finding two-word food items were identified (e.g., cheese sandwich, chocolate biscuit)	Search was improved to match on more than one search term
Portion-size options for generic foods were challenging for users as only one option could be selected (e.g., for generic orange juice only a 200ml glass could be selected)	Significant work was done on providing a range of appropriate portion-size options for generic food items
“Bug” in final nutrient summary output which lead to miscalculation of total macronutrients displayed	“Bug” fixed and nutrient summary output, which was checked against a sample of manually coded diaries
Recipe builder was not intuitive and difficult to use	Text in the recipe builder was reworded to make the flow easier to follow
People disliked having to add in meal slot details each time for each individual food item	Meal slots were defaulted to previously selected so only needs to be clicked when moving to a different meal slot rather than each individual food. Drag-and-drop between meal slots enabled. Ability to select meal slot on left side of diary in advance and then add foods was added
Long food descriptors were not displayed in full	Text was wrapped so that entire food description displayed
It was not clear on final screen that the food diary has been completed	Text was added to confirm that diary is complete and safe to close browser

**Table 4:** Sample characteristics by age group and system usability scale (SUS) scores for participants completing usability testing of the beta and live versions of myfood24.

Age group	Adolescents (11–18 Years)		Adults (19–64 Years)		Older Adults (≥65 Years)	
Sample characteristic	Beta	Live	Beta	Live	Beta	Live
N	14	70	8	20	1	4
Female (%)	57	50	75	74	100	75
System Usability Scale score (Median (IQR))	66 (20)	73 (22)	68 (40)	80 (25)	38	29 (63)
Technology confidence ^(a)^ (Median, (IQR))	9 (1)	9 (2)	8 (1)	8 (2)	8	3 (2)

^(a)^ Self-rated based on a 10-point Likert scale (1 = not confident at all; 10 = extremely confident).

### 3.5. Stage 5: Launch of Live Site

**Figure 4:**
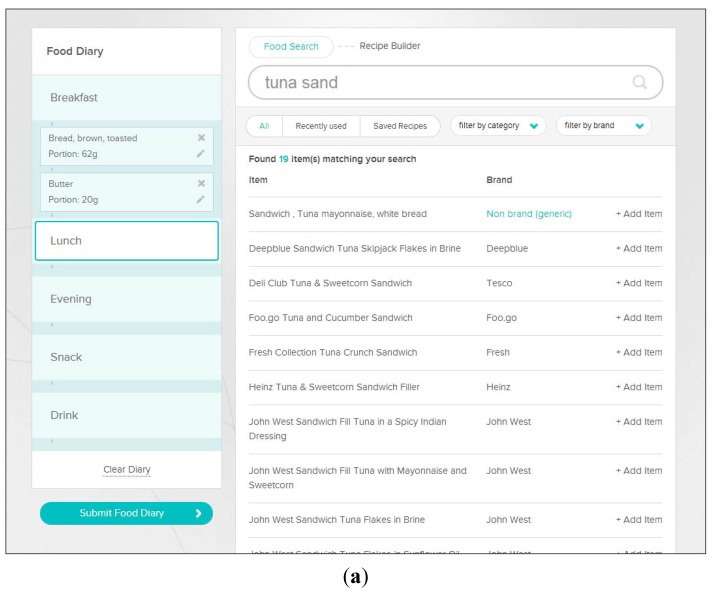

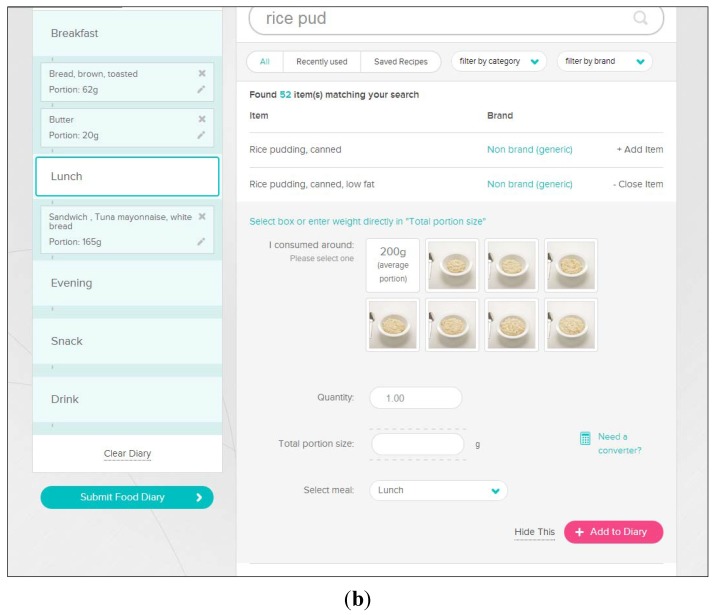
(**a**) Screenshot to show searching and logging items consumed using myfood24. (**b**) Screenshot to show estimating portion sizes using myfood24.

Figure 4a,b are examples of the myfood24 participant interface. To assess usability before the tool was launched as live for the validation study, a further round of usability testing took place.

The adolescent participants were recruited from two high schools in different areas of Leeds. The adult participants were recruited from a convenience sample in London and Leeds. Older people were recruited from a convenience sample in Leeds. Anyone who had used myfood24 before, or had been involved with the development in any way, was not eligible to take part. The participants were required to report their food intake over the previous 24-h using myfood24. Basic demographic data were collected and the SUS was administered. Participants gave feedback relating to specific features of myfood24.

The live version of myfood24 was tested by 70 adolescents, 20 adults and four older adults. Attempts were made to contact 10 older adults but only four responded. The sample characteristics, SUS score and self-rated technology confidence score of those who conducted usability testing of the beta and live version of myfood24 can be found in Table 4. Qualitative results relating to testing of the live version of myfood24 with adults can be found in Table 5. A noteworthy result from this stage of testing is the length of time it took adult participants to complete their 24-h dietary recalls. In the sample of 24 adults using the live site, the mean completion time was 19 (SD: 7) minutes.

Median SUS in adolescents and adults were moderate for the beta version of myfood24 (adolescents median SUS = 66, (interquartile range) IQR = 20; adults median SUS = 68, IQR = 40) and good for the live version (adolescents median SUS = 73, IQR = 22; adults median SUS = 80, IQR = 25). Among older adults the median SUS score was poor for both the beta (median SUS = 38) and live version (median SUS = 29, IQR = 63).

**Table 5:** A summary of qualitative findings relating to specific areas of the live version of myfood24 with a sample of adult users.

Features of myfood24	Answer % (*n*/*n*)	Notes
MY PROFILE-Are you able to load the webpage and enter your details successfully?	Yes—100 (20/20)	All able to enter details
GETTING STARTED-Have you read the instructions and is the language appropriate and easy to understand?	Yes—95 (18/19)	Mostly straightforward; 1 person was not sure how to complete the diary page
MAKE A LIST-Did you use the “Make a list” function. If so, did you find it easy to use?	Used—40 (8/20) Easy—75 (3/4)	Some did not notice feature
SEARCH-Was the search function easy to use? Could you find items easily?	Easy—89 (17/19) Find items—29 (4/14)	Some brands not listed and some lists too long
PROMPTS-Did you find any prompts that came after you entered certain foods to be helpful? Did you respond to the prompts?	Helpful—94 (17/18) Respond—67 (8/12))	Useful overall
PORTION SIZE ENTRY-Did you find it easy to understand how to enter portion size? Did you find it confusing to have both pictures *and* grams displayed on the screen?	Easy—89 (16/18) Confusing—21 (3/14))	Pictures found to be helpful
RECENTLY USED ITEMS-Could you find and use the “Recently used items” list?	Used—29 (5/17)	Not widely used but no negative comments
RECIPE BUILDER-Was the recipe builder easy to use?	Yes—88 (7/8)	Convenient; some found it confusing and time investment required
SUBMITTING FOOD DIARY-Could you submit your food diary?	Yes—85 (17/20)	Some had error messages on submission
TIME TAKEN TO COMPLETE-How long did it take you to complete your intake and submit your diary?	Mean (SD) 19 (7) minutes	
HELP-Did you use the help text or the help video? If so, did you find it useful?	Used—5 (1/20) Useful—100 (1/1)	

#### Availability of myfood24

In order that myfood24 remains up-to-date with respect to new products or reformulations of existing foods, the tool will be hosted and maintained by the University of Leeds. The tool has been developed such that it can be made available to researchers worldwide through a unique login. For enquiries relating to use of myfood24 in research please contact myfood24@leeds.ac.uk. myfood24 has a demonstration feature so it is possible to try the front-end (participant side) of the tool by visiting: www.myfood24.org and using the “demo” button.

## 4. Discussion

myfood24 is the first online 24-h dietary recall tool targeted for use with the UK population. The findings from the focus groups were used to inform the specifications of myfood24. After development of a beta version, usability testing was conducted to further refine the tool. The focus groups highlighted that an overriding requirement from potential system users was a system that is quick and easy to use. During the development, we balanced the needs of the researchers to collect detailed and accurate dietary assessments with the users’ desires to spend minimal time using the tool. To reduce the time taken to complete the food intake, we therefore chose not to pursue the detailed AMPM method. Within myfood24, users are asked to move through as few screens as possible to complete food recalls and “pop-ups” and prompts are limited. However, the myfood24 tool has retained some aspects of the AMPM, with an optional quick-list as the first pass, detailed food search, forgotten-item prompts for commonly-forgotten foods, and final review before submission. Although checks and final checks are built into the system most pages within myfood24 can be reached within a few clicks. This design approach was adopted based on the desires of focus group participants and experience with the MMM app (see Section 3.1). Thus, although myfood24 does not fully embrace the AMPM method, the strengths of this method have still been applied.

To ensure the system is intuitive and easy to use, a new food composition database was developed for the tool, and consideration has gone in to refining the database search function [20]. Whilst the existing British food composition tables contain ~3500 generic food items [17], the food database developed for myfood24 contains ~45,000 UK branded and generic foods with their associated pack and portion sizes. The median adult SUS score of the myfood24 live version at 80/100 is in the “good” range for websites and compares favorably with other behavior-assessment websites, such as a smoking cessation website rated at 67 [31] and a physical activity website rated at 73 [32].

### 4.1. Strengths and Challenges

A major strength of myfood24 is that it is the first UK online 24-h dietary assessment tool aimed at the UK adult population and incorporates a novel and extensive food composition database to generate instant nutrient values without the need for coding. The system has been informed by the views of three different age groups (adolescents, adults and older adults) to be user-friendly among a wide range of people. The flexible researcher website permits a range of study types and personalization of information presented to participants. A strength of the myfood24 development process has been the iterative approach which has facilitated the creation of a user friendly tool. The usability testing discussed in this study has shown myfood24 to be highly rated in adolescents and adults.

A limitation of the development process was the small number of older adults who were involved in focus groups and undertook testing of the beta and live versions of the system. Ten older adults were contacted at both testing points (beta and live), however at the beta stage only one was able to complete the tool and only four (with low computer literacy) were able to complete the final version. The SUS for those older adults who did complete the tool was very low in comparison to the other two age groups, which were considerably higher. There are a number of factors such as general lack of technical knowledge, fine motor control issues and hearing and vision loss which can affect an older adult’s ability to use the Internet [33]. These factors may have affected their ability to use myfood24. It is also worth noting that self-rated confidence in using technology was much lower for the older adults (median *=* 3, IQR = 2 for the live site) than the other two age groups (adolescents median *=* 9, IQR = 2; adults median *=* 8, IQR = 2), which may have influenced engagement with myfood24. The low SUS score is in line with the observation that SUS scores generally decrease with age of the user [34]. Further research is planned to investigate what changes could be made to myfood24 to improve its usability with older adults. There is a facility for a researcher to “take control” of the recall so that it can be administered over the phone or face-to-face by an interviewer. This might be a more suitable option for using the tool with older people.

Given the complexity and detailed requirements of the tool, barriers encountered during working with an external software company included separate geographical location, communication and effective project management. This was overcome in the later stages with additional face-to-face meetings and introduction of financial milestones. This has been identified as a common difficulty when nutrition researchers work with external software companies [35]. Maintaining an up-to-date food composition database will be an ongoing challenge given that food and beverage manufacturers regularly reformulate products or introduce new products to the market. Ongoing funding will therefore be necessary to host and maintain the website.

### 4.2. Planned Future Work

myfood24 is being validated in a sample of adults against reference measures of 3 researcher-administered 24-h dietary recalls over 3 months and 3 blood and urine collection biomarker assessments. The tool will be piloted in (1) a sample of the UK Women’s Cohort Study, which includes 35,000 women [36]; (2) in a clinical sample of women with gestational diabetes mellitus; and (3) within the Airwaves Health Monitoring Study cohort at Imperial College London [37]. Furthermore, a relative validity study has been conducted among adolescents (11–18 years old) to compare myfood24 *vs.* interviewer-administered 24-h dietary recall.

A new feature will be added to incorporate a range of different food composition tables from different nationalities. This will allow the researcher to determine which food composition databases will be displayed in the myfood24 food search so that databases for different countries can be made available with relative ease. Furthermore, regular maintenance will be performed to the system to ensure that the food composition database remains up to date.

## 5. Conclusions

Myfood24 is the first online multiple-pass 24-h dietary assessment tool for the UK population. Foods are selected from a new comprehensive food composition database, which has been specifically designed for and built into the tool. Focus groups undertaken with three age groups have informed the development of the tool, and usability testing has been conducted with beta and live versions of myfood24 to facilitate an iterative development process. Usability testing indicated that myfood24 is suitable for use in UK adolescents and adults. For enquiries relating to use of myfood24 please contact myfood24@leeds.ac.uk.

## Supplementary Files

Supplementary File 1
