# Supplementary material for: Development of a UK Online 24-h Dietary Assessment Tool: myfood24
Source: Nutrients. 2015 May 27;7(6):4016–32. doi: 10.3390/nu7064016 (PMC4488770; doi:10.3390/nu7064016)
Supplement: Supplementary File 1 [file nutrients-07-04016-s001.docx]

Supplementary Material


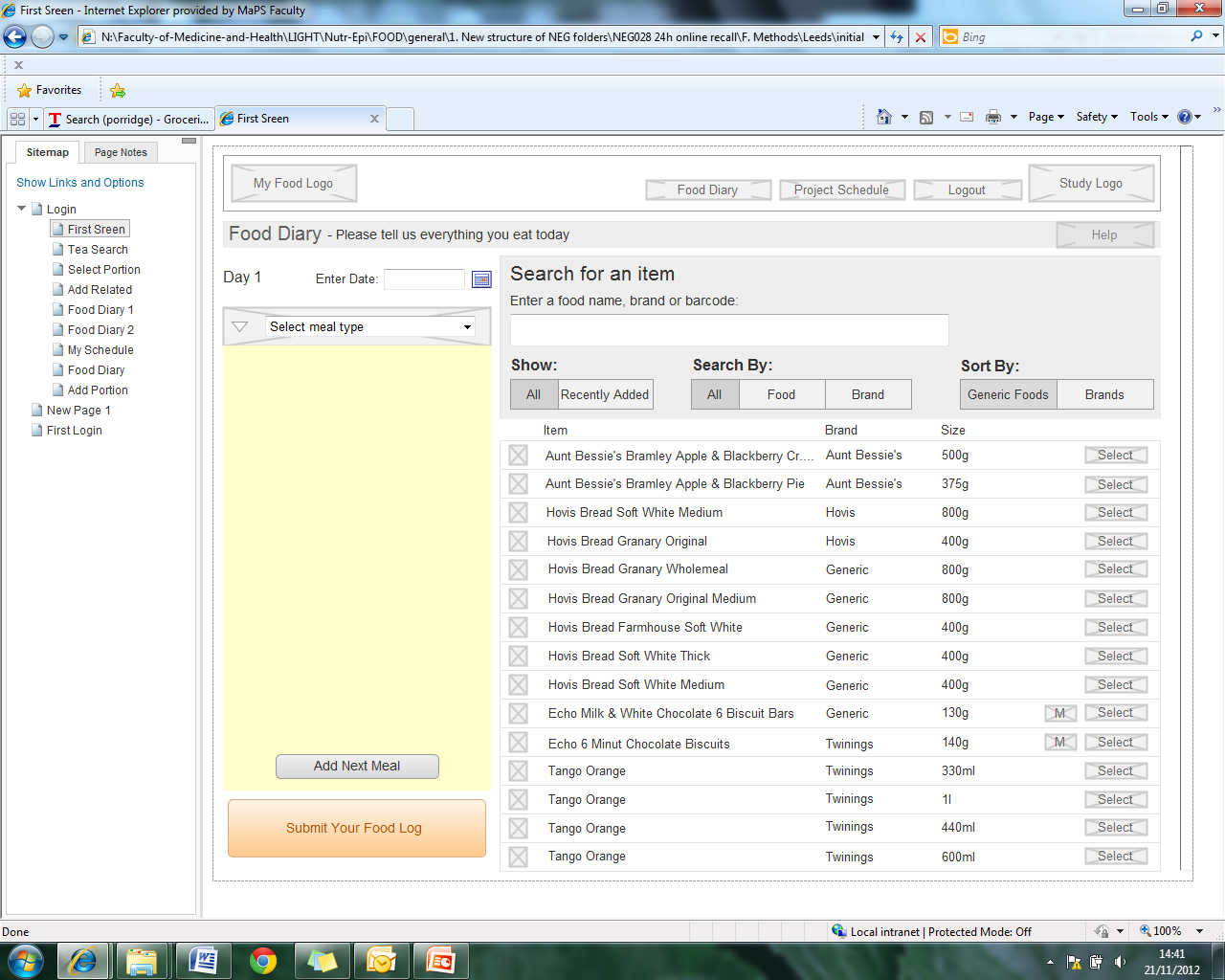


**Figure S1.** *Cont.*


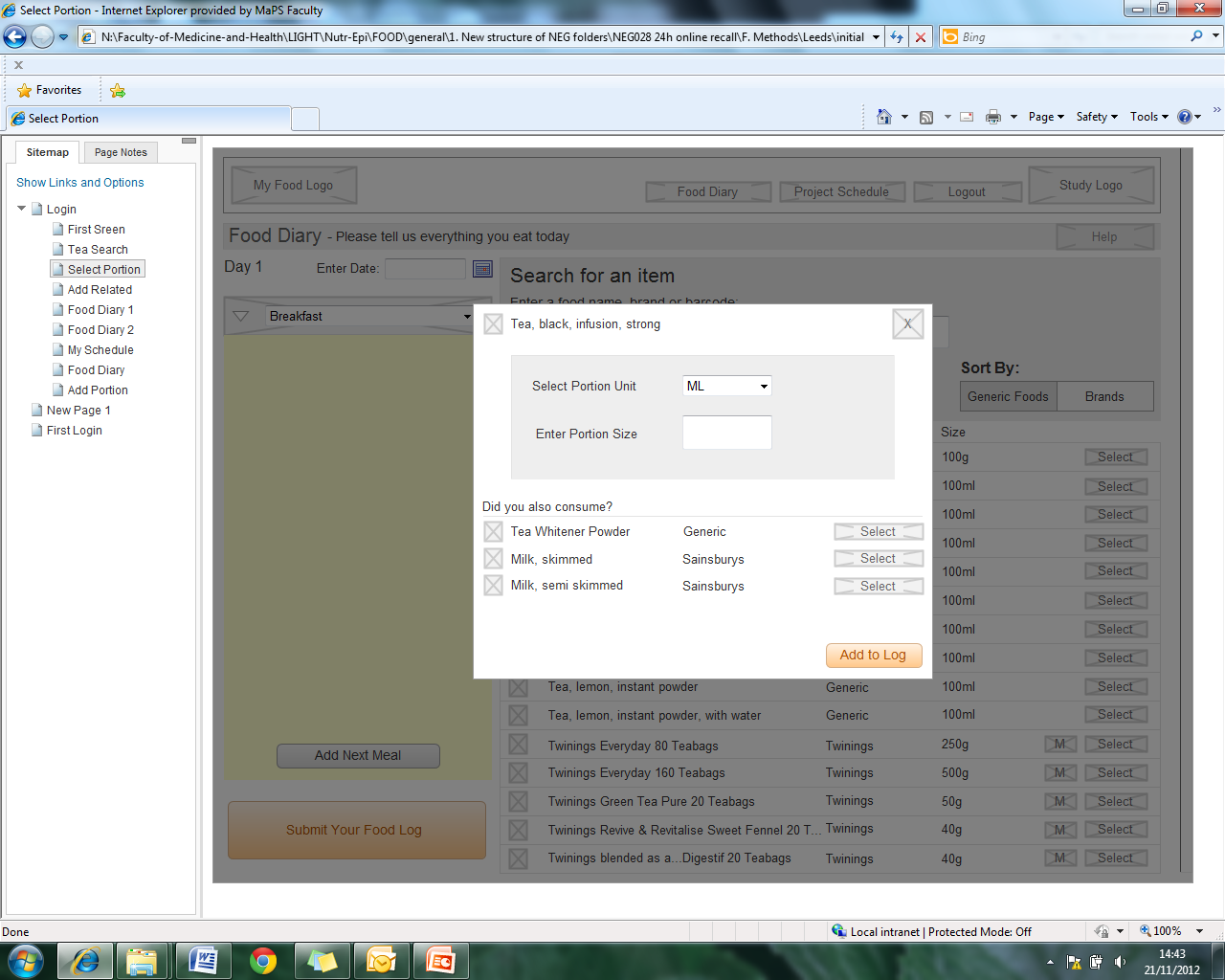


**Figure S1.** (**a**) Example of initial static click-through wireframe of myfood24 (food diary page)–clay model; (**b**) Example of initial static
click-through wireframe of myfood24 (food portion selection).

| **** | **** |
| --- | --- |
| (**a**) | (**b**) |

**Figure S2.** (**a**) Example of options to set up a project in the researcher area of myfood24; (**b**) Example of options to customise text displayed to the participant in the researcher area of myfood24.

© 2015 by the authors; licensee MDPI, Basel, Switzerland. This article is an open access article distributed under the terms and conditions of the Creative Commons Attribution license (http://creativecommons.org/licenses/by/4.0/).
